# Enhanced mechanical performance of biocompatible hemicelluloses-based hydrogel via chain extension

**DOI:** 10.1038/srep33603

**Published:** 2016-09-16

**Authors:** Xian-Ming Qi, Ge-Gu Chen, Xiao-Dong Gong, Gen-Que Fu, Ya-Shuai Niu, Jing Bian, Feng Peng, Run-Cang Sun

**Affiliations:** 1 Key Laboratory of Lignocellulosic Chemistry, Beijing Forestry University, 100083, China; 2 College of Life Science, Agricultural University of Hebei, Baoding, Hebei, 071001, China; 3 State Key Laboratory of Pulp and Paper Engineering, South China University of Technology, Guangzhou 510640, China

## Abstract

Hemicelluloses are widely used to prepare gel materials because of their renewability, biodegradability, and biocompatibility. Here, molecular chain extension of hemicelluloses was obtained in a two-step process. Composite hydrogels were prepared via free radical graft copolymerization of crosslinked quaternized hemicelluloses (CQH) and acrylic acid (AA) in the presence of crosslinking agent *N,N’*-methylenebisacrylamide (MBA). This chain extension strategy significantly improved the mechanical performance of the resulting hydrogels. The crosslinking density, compression modulus, and swelling capacities of hydrogels were tuned by changing the AA/CQH and MBA/CQH contents. Moreover, the biocompatibility test suggests that the hemicelluloses-based hydrogels exhibited no toxicity to cells and allowed cell growth. Taken together, these properties demonstrated that the composite hydrogels have potential applications in the fields of water absorbents, cell culture, and other functional biomaterials.

Hydrogels are three-dimensional networks of crosslinked molecules and the vast majority of their mass consists of water. However, they still exhibit solid-like mechanical properties^1^,^2^,^3^,^4^ Hydrogels attract more and more attention due to their potential use in a wide range of applications, such as superabsorbent materials^5^, drug delivery^6^,^7^, biosensors^8^,^9^, tissue engineering^10^,^11^, matrix chemistry^12^, and biology^13^,^14^. Polysaccharides have great advantages in the fabrication of hydrogels because of their economical, biocompatible, nontoxic, and biodegradable properties^15^,^16^,^17^,^18^. Hemicelluloses, a kind of renewable resources, are the second most abundant polysaccharides in nature, making up about 20–35% of lignocellulosic biomass. Potential applications of hemicelluloses and their derivatives are being investigated in many fields, such as the preparation of hemicelluloses-based hydrogels^19^,^20^. The chemical modification of hemicelluloses provides a potential route to prepare value-added materials with unique properties that can increase the utility of these biopolymers^21^,^22^,^23^. A series of hemicelluloses-based hydrogels were synthesized by introducing functional monomers with unsaturated bonds to the backbone of hemicelluloses and chemical crosslinking of the modified hemicelluloses. These fabricated hydrogels presented good temperature sensitivity, biodegradability, nontoxicity, and controllable swelling capacity^24^,^25^,^26^,^27^. Xylan-rich hemicelluloses-graft-acrylic acid (AA) ionic hydrogels were responsive to pH, salt, and organic solvents^28^. A porous hydrogel prepared by graft copolymerization of AA and hemicelluloses can absorb heavy metal ions^29^. Temperature sensitive hemicelluloses-based hydrogels were fabricated by UV photo crosslinking of hemicelluloses and *N*-isopropylacrylamide^24^. These hemicelluloses-based hydrogels are promising biomaterials with potentially widespread applications, and a series of other natural and biomimetic hydrogels with novel functions have been designed^30^,^31^,^32^,^33^. However, the mechanical properties of hemicelluloses-based hydrogels remain weak and the water swollen hemicelluloses based hydrogels are brittle. In addition, there are few current studies on the mechanical properties of hemicelluloses-based hydrogels. Due to hemicelluloses with a low molecular weight and a degree of polymerization in the range of 80–450, great efforts have been made to improve the mechanical properties of hemicelluloses-based hydrogels without impairing their swelling capacities^34^. In this study, molecular chain extension of hemicelluloses was obtained in a two-step process. First, quaternary ammonium groups were introduced to hemicelluloses by etherification, and then the molecular chains of the hemicelluloses were extended via a crosslinking reaction. This scheme should be a practicable approach to improve the mechanical properties of hemicelluloses-based hydrogels.

Acrylic acid is an important monomer, and is widely used to prepare functional hydrogels. The incorporation of AA in the hydrogel network is generally used for superabsorbent materials and to promote the removal of heavy metal ions or dyes^35^,^36^. Here, we describe a strategy for the formation of hemicelluloses-based hydrogels. The extended molecular chains of hemicelluloses were considered as the backbone, and the hydrogel network was formed by crosslinking AA monomers based on this backbone. After synthesis, the properties of the composite hydrogels were investigated.

## Results and Discussion

### Reaction Mechanism

**Figure 1:**
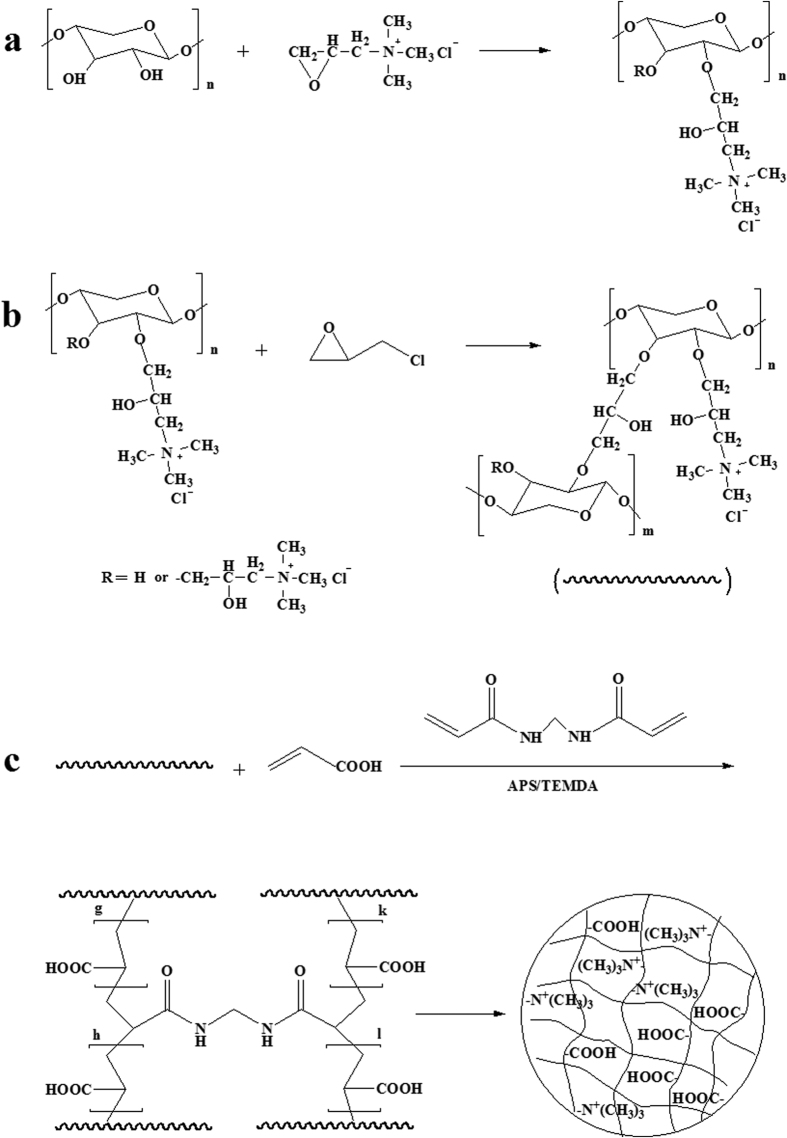
The synthetic process of QH (**a**), CQH (**b**), and composite hydrogels (**c**).

The composite hydrogels were prepared between crosslinked quaternized hemicelluloses (CQH) and AA by free radical graft copolymerization in the presence of crosslinking agent *N,N’*-methylenebisacrylamide (MBA) and a redox initiator system of ammonium persulfate (APS) and *N,N,N’,N’*-tetramethylethylenediamine (TEMED). Diverse weight ratios of AA/CQH and MBA/CQH were used to explore effects on the network structure of the composite hydrogels. The crosslinking process of graft copolymerization of AA onto hemicelluloses in the presence of MBA is shown in Fig. 1c. Briefly, sulfate anion radical generated from heated APS and abstracted hydrogen from the hydroxyl group of hemicelluloses form alkoxy radicals, resulting in active centers on the hemicelluloses backbone to radically initiate polymerization. Since the crosslinking agent (MBA) was present in the system, it led to crosslinking of the copolymer network, preventing dissolution of the hydrophilic polymer chains in an aqueous environment. In this way, free radical graft copolymerization was used to generate a series of hemicelluloses-based composite hydrogels with different weight ratios of AA/CQH and MBA/CQH. The composition of the fabricated is shown in Table 1.

### FT-IR Analysis

**Figure 2:**
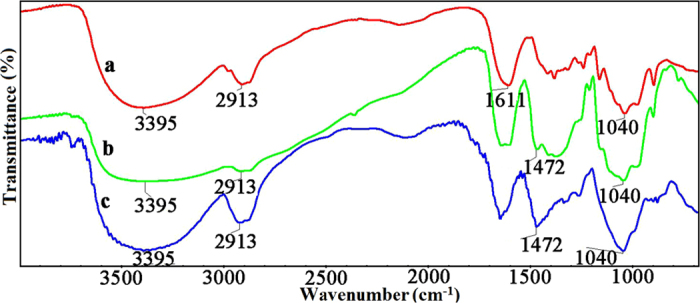
FT-IR spectra of hemicelluloses (**a**), QH (**b**), and CQH (**c**).

FT-IR spectroscopy was performed to monitor the crosslinking process and the resulting products. Figure 2 shows the FT-IR spectra of hemicelluloses (spectrum a), QH (spectrum b), and CQH (spectrum c). In Fig. 2a, the strong broad absorption peak at 3395 cm^−1^ and the band at 2913 cm^−1^ are attributed to the stretching vibration of -OH and symmetric vibration of C-H, respectively. The band at 1611 cm^−1^ is assigned to the bending mode of absorbed water^37^. The prominent absorption peak at 1040 cm^−1^ is originated from the C-O-C stretching vibration^38^. In the FT-IR spectra of QH (spectrum b) and CQH (spectrum c), the absorption bands at 3395, 2913, and 1040 cm^−1^ which respectively belong to the vibration of -OH, C-H, and C-O-C, are consistent with the peaks of hemicelluloses. In addition, a new absorption peak at 1472 cm^−1^ appeared in both QH and CQH, which originates from the bending vibration of -CH_3_ and -CH_2_ in quaternary ammonium group, indicating that the resulting product was obtained by etherification reaction between hemicelluloses and 2,3-epoxypropyltrimethyl ammonium chloride (ETA)^39^. Compared with spectra a and b, the increased intensity of the bands at 1472 and 1040 cm^−1^ at spectrum c indicate the molecular chain extension of hemicelluloses.

**Figure 3:**
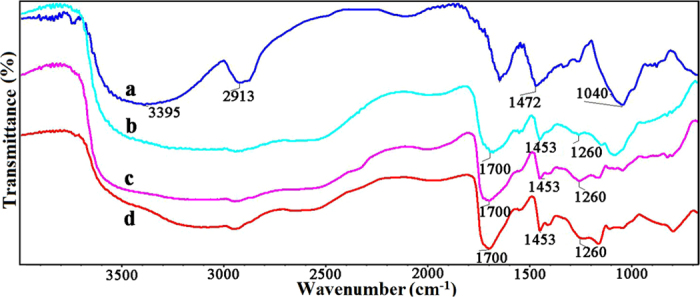
FT-IR spectra of CQH (**a**), H-0.8 (**b**), H-1.2 (**c**), and H-1.6 (**d**).

The FT-IR spectra of CQH, H-0.8, H-1.2, and H-1.6 are shown in Fig. 3. The composite hydrogels were prepared by graft copolymerization of AA onto hemicelluloses in the presence of MBA. Compared with the two absorption peaks at 3395 and 2913 cm^−1^ of hemicelluloses, the vibrational bands at a range of 3500~2900 cm^−1^ in the spectra of H-0.8, H-1.2, and H-1.6 are assigned to the vibration of -OH of polyacrylic acid, which become broad and cover these two peaks of hemicelluloses^40^. In spectra b, c, and d, the new peaks at 1700 and 1453 cm^−1^ are related to the stretching vibration of C=O and -CH of quaternary ammonium group, respectively. The band at 1260 cm^−1^ is due to the stretching vibration of C-N. These results indicate that AA monomers were successfully grafted onto the backbone of CQH in the presence of MBA.

### Swelling Behaviors and Morphological Analysis of Hemicelluloses-Based Hydrogels

**Figure 4:**
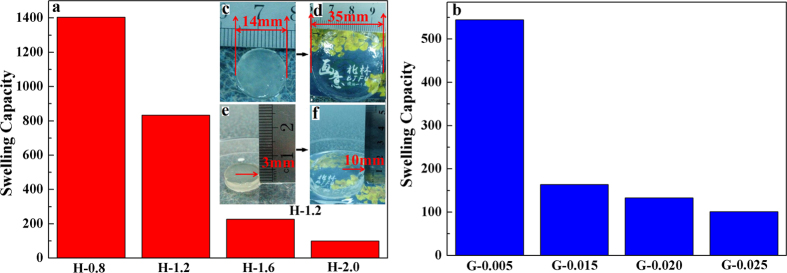
Swelling capacity of hydrogels with different concentrations of AA (**a**) and MBA (**b**).

The swelling capacity is an important indicator of hydrogel crosslinking density and internal structure, describing the amount of absorbed water in the hydrogel network^41^,^42^. The swelling capacities of hydrogels are shown in Fig. 4a,b. The composite hydrogels consist of highly negatively charged carboxylic groups, producing internal electrostatic repulsion that leads to structural expansion and high swelling capacity of the hydrogel^28^. The state of the composite hydrogel H-1.2 before and after reaching swelling equilibrium was determined. Figure 4c,e are the original hydrogels before swelling. The diameter and height of the original thin cylindrical hydrogels are 14 and 3 mm, respectively. The photos of hydrogels after reaching swelling equilibrium are shown in Fig. 4d,f. There were significant changes in the appearance of the hydrogel, and the diameter and height of the hydrogel reached 35 and 10 mm, respectively. The volume of the swollen hydrogel was about 20.8 times greater than that of the original hydrogel. In addition, as shown in Fig. 4d, the swollen hydrogel had excellent transparency. The pattern covered with the hydrogel could be seen clearly even at a height of 10 mm.

**Figure 5:**
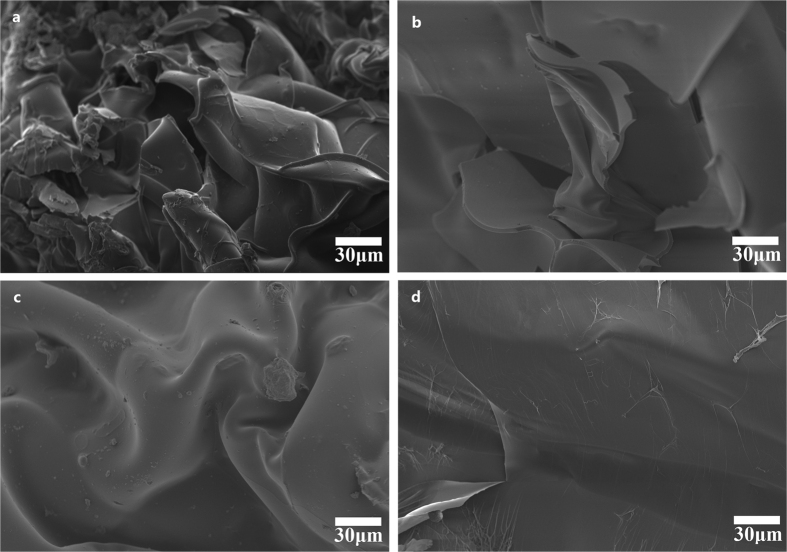
SEM images of hydrogel H-0.8 (**a**), H-1.2 (b), H-1.2 (**c**), and H-2.0 (**d**).

Figure 4a shows the swelling ratio of hydrogels from H-0.8 to H-2.0. With the AA/CQH weight ratio increasing from 8:1 (H-0.8) to 20:1 (H-2.0), the swelling ratio decreased from 1405 to 100 g/g. This result suggested that the hydrogels have less expanded network structures inside hydrogels with increasing amount of AA. In other words, a higher amount of AA monomers in the preparation of composite hydrogels resulted in a higher crosslinking density and a denser network structure. This is consistent with the scanning electron microscopy (SEM) images of hydrogels in Fig. 5. The interior crosslinked structure of the composite hydrogels can be characterized by a SEM. The SEM images of the hydrogel H-0.8, H-1.2, H-1.6, and H-2.0 at magnifications of 500× are shown in Fig. 5. All of the hydrogels showed different extents of fold on their surfaces, which may indicate regions of water absorption and retention. As shown in Fig. 5a (H-0.8, AA/CQH weight ratio of 8:1), the hydrogel H-0.8 showed a network with more lamellar structures and folds compared with other four hydrogels. These lamellar structures and folds improved the specific surface area of hydrogel, allowing greater water absorption and improving swelling capacity of hydrogels. Furthermore, as shown by comparison of the images from image a (H-0.8) to image d (H-2.0), an increased amount of AA monomers led to a gradual decrease in the lamellar structures and folds. These results indicated that the regions of water absorption and retention decreased as the amount of AA monomers increased, in agreement with the swelling capacity results in Fig. 4a.

**Figure 6:**
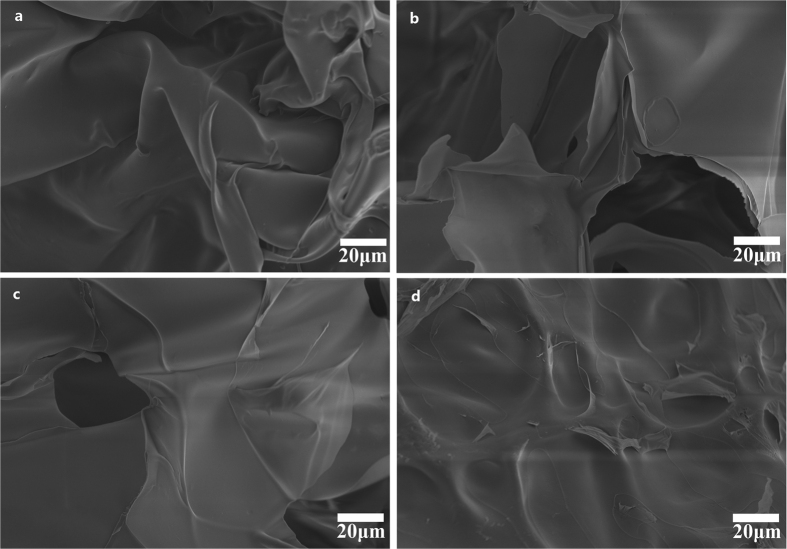
SEM images of hydrogel G-0.005 (**a**), G-0.015 (**b**), G-0.020 (**c**), and G-0.025 (**d**).

The effects of MBA/CQH ratios on the swelling capacity of hydrogels in distilled water are demonstrated in Fig. 4b. As the MBA/CQH ratio increased from 0.05 to 0.25, the swelling capacity of hydrogels decreased gradually from 544 to 101 g/g. This indicated that increased amounts of crosslinking agent MBA reduced the swelling ratio of composite hydrogels. The crosslinking agent MBA played an important role in three-dimensional network structure of the hydrogels. If there was an insufficient amount of MBA, the hydrogel was unable to form a good three-dimensional network with low crosslinking density, resulting in increased solubility of the hydrogel, increased ease of deformation, and strength loss. In contrast, with an excess amount of MBA, the crosslinking density of hydrogel is increased and there is a less developed network inside the hydrogel, which diminished swelling capacity of hydrogel. The SEM images of hydrogel G-0.005 (a), G-0.015 (b), G-0.020 (c), and G-0.025 (d) at magnifications of 750× are shown in Fig. 6. There were many folds on the surface of G-0.005, and these folds could improve the specific surface area of the hydrogel, improving the swelling behavior. The folds on the hydrogels surface decreased gradually as the MBA amount increased from G-0.005 (Fig. 6a, MBA/CQH ratio of 0.05:1) to G-0.025 (Fig. 6d, MBA/CQH ratio of 0.25:1). As shown in Fig. 6d, the surface of the hydrogel G-0.025 was very smooth with minimal folds. Therefore, the amounts of folds on the hydrogel surface determine the swelling capacity of the hydrogels.

### Mechanical Properties

**Figure 7:**
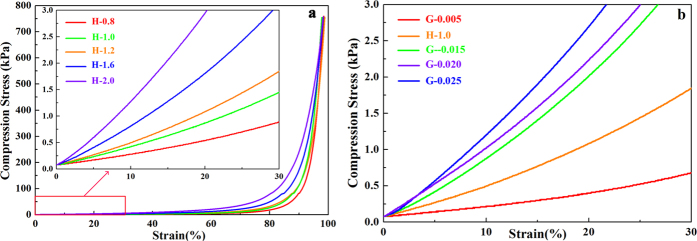
Compression stress-strain curves of hydrogels with different concentrations of AA (**a**) and MBA (**b**).

The cylindrical composite hydrogel samples were tested on a compressive tester to record the compression stress-strain curves. The compression stress-strain curves of hydrogels H-0.8, H-1.0, H-1.2, H-1.6, and H-2.0 are shown in Fig. 7a. All of the hydrogels were able to tolerate compression greater than 95% and exhibited partially rubber-like properties. The compression stress of the composite hydrogels increased slightly with increasing of deformation, and all the hydrogel samples showed similar curves despite of the different concentrations of AA. This suggested that these composite hydrogels exhibited high toughness and elasticity because of the three-dimensional network. The maximum compression stress of the five hydrogels reached about 750 kPa when the strain reached about 95%, which were superior or comparable to those reported for other hydrogels based on pure hemicelluloses. For example, the previous report on the compression stress of the pure hemicelluloses-based hydrogel was only about 38.6 kPa when the hydrogel was totally compressed^28^. Furthermore, no fracture or break was observed in these five hydrogel samples at that stress level. This clearly indicated that the molecular chain extension of hemicelluloses was beneficial to enhance the mechanical strength of the hemicelluloses-based hydrogels. When the strain exceeded 80%, the overall compression stress-curves rose sharply. Besides, when the different compression stress-curves were in the same strain, the compressive modulus and strength increased with increased AA concentration.

To further investigate the effects on the compression modulus with different concentrations of AA and MBA, strain of 0–30% was selected to better demonstrate the compression stress-strain curves of composite hydrogels. The compression modulus is the ratio of stress to strain, and it is an important parameter of compressive deformation. A greater value of compression modulus indicates more stiffness of the hydrogel, and a lesser value indicates that the hydrogels are flexible and easy to be compressed. As shown in Fig. 7a, the compression modulus of the hydrogel increased with increasing concentration of AA, which indicated that the higher the amount of AA in the hydrogel, the more the stiffness of the hydrogel. It was presumed that increasing the amount of AA would promote the crosslinking density and tighten the network structure. Similar results were found in the samples of G-0.005 to G-0.025 with increasing concentration of MBA, as shown in Fig.7b. A higher concentration of MBA produced a higher crosslinking density and decreased the spaces between the copolymer chains, resulting in a hydrogel network structure that resists compression. Overall, these findings demonstrate that the crosslinking density can be tuned from low to high by changing the ratios of AA/CQH and MBA/CQH, which determine the compression modulus and swelling capacity of hydrogels. Therefore, these analyses of the compression modulus with different concentrations of AA and MBA were consistent with the swelling capacity results.

**Figure 8:**
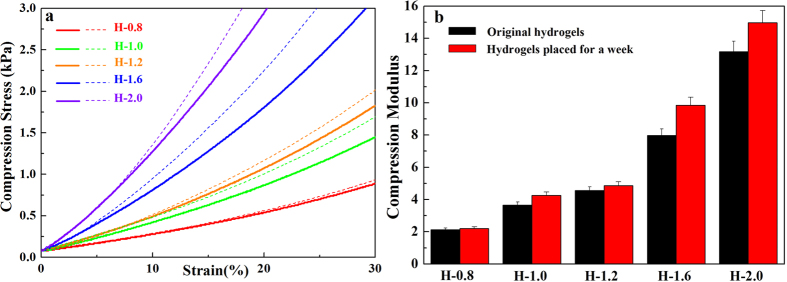
Compression stress-strain curves of original (solid lines) and 7-day hydrogels (dashed lines, (**a**). Compression modulus of original and 7-day hydrogels with different concentrations of AA (**b**).

The patterns of compression stress-strain do not change linearly but are smooth curves because hydrogels are soft materials. Therefore, the compression modulus of the hydrogel continued to change as strain increased. However, as shown in Fig. 8a, the flat curve can be treated as a straight line within a narrow range of strain. The compression stress-strain curves of the original hydrogels and the hydrogels having been placed for a week are demonstrated in Fig. 8a. Solid lines represent the strain curves of the original hydrogel, and the dashed lines represent the 7-day hydrogels. The curves of the 7-day hydrogels were higher than the curves of the original hydrogels in every case, which was explained that the compression modulus became stronger as the storage time went by. As shown in Fig. 8b, the compression modulus of the hydrogel was further quantified to determine the influence of storage time on hydrogel at the strain of 15%. The 7-day hydrogels showed a higher compression modulus in contrast to the original hydrogels due to a change in moisture content, as shown in Table 2. The higher water content can promote elasticity of the composite hydrogels^43^. Higher water content indicated more water in the spaces between the copolymer chains in the hydrogel, which was beneficial for the flexibility of hydrogel. Interestingly, the compression modulus difference between the 7-day hydrogels and the original hydrogels increased with increasing concentration of AA. The compression modulus difference of H-0.8 was 0.07 kPa, but the compression modulus difference of H-2.0 was 1.81 kPa. As shown in Table 2, the change of compression modulus was consistent with the change of water content, and the higher water content improved hydrogel flexibility. The water retention capacity of hydrogels decreased with increased AA concentration, because the higher AA concentration increased the crosslinking density for a denser network structure with less space between copolymer chains, allowing the release of water molecules.

### Biocompatibilities of the Hydrogels

**Figure 9:**
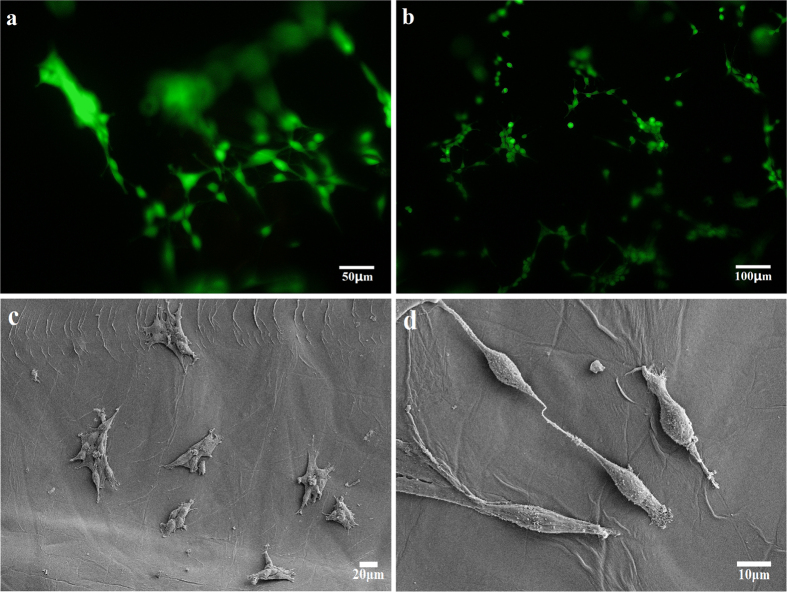
Fluorescent images of preosteoblasts cultured on hydrogel H-1.2 for 24 h (**a**) and 36 h (**b**); and SEM images at magnifications of 200× (**c**) and 1000× (**d**).

To further evaluate the biocompatibility of the composite hydrogels, preosteoblasts were cultured on the hydrogels. The fluorescent images of preosteoblasts stained with dye and SEM images of preosteoblasts without staining treatment are shown in Fig. 9. Figure 9a,b are the fluorescent images of preosteoblasts cultured on hydrogel H-1.2 for 24 and 36 h, respectively. The seeded preosteoblasts could adhere, spread, and proliferate well on the composite hydrogels. As can be seen from the fluorescent images, preosteoblasts grew well on the hydrogels and exhibited a high cell density, indicating that the hydrogel could serve as a matrix for cell adhesion and proliferation. The SEM images of preosteoblasts cultured on the hydrogel for 24 h were used to evaluate biocompatibility, as shown in Fig. 9c,d. The morphology of the preosteoblasts as observed from the SEM images indicated that the fabricated hydrogels have good biocompatibility. There were many layered folded structures inside the hydrogel that may absorb extracellular protein efficiently and provide space for cell adhesion and spreading, resulting in good biocompatibility. Therefore, the fabricated hydrogels could be used as a biocompatible matrix, allowing potential applications in the field of tissue engineering.

## Materials and Methods

Hemicelluloses were extracted from bamboo (*Phyllostachys pubescens*) holocellulose by the alkaline extraction method^44^. Sodium hydroxide, epichlorohydrin (ECH), AA, and ethanol were purchased from Beijing Chemical Works. 2,3-epoxypropyltrimethyl ammonium chloride (ETA) was obtained from sigma-aldrich Co., Ltd. APS was purchased from Xilong Chemical Co., Ltd. TEMED was obtained from AMRESCO. MBA was purchased from Chengdu Gray West Chemistry Technology Co., Ltd. All reagents mentioned above were directly used without further purification.

### Preparation of Quaternized Hemicelluloses (QH)

QH were synthesized in accordance with twice alkalization method described by Ren *et al*.^45^. Typical procedures for the reactions between ETA and hemicelluloses in aqueous alkaline systems are as follows. Hemicelluloses (0.99 g) were dissolved in 30 mL of distilled water in a three-necked flask fitted with a reflux condenser with vigorous mechanical stirring at 60 °C for 30 min. Then the solution was cooled to 30 °C. The first proportion of aqueous sodium hydroxide (4.0 mL, the molar ratio of NaOH to ETA, 0.5) was added to alkalize for 20 min, and then the mixed solution was heated to 60 °C again. ETA (0.03 mol, the molar ratio of ETA to anhydroxyloses units in hemicelluloses, 2.0) was added dropwise into the mixture within 20 min, and the solution continued to be stirred for 30 min. The rest proportion of aqueous sodium hydroxide (6.0 mL) was added into the solution, and the reaction mixture was additionally stirred for 5 h at 60 °C. The resulting hemicelluloses derivatives were obtained by ethanol precipitation. The QH precipitates were filtered off and washed thoroughly with ethanol for removing residual reagents. The purified product was first air dried for 12 h and then further dried in an oven for 24 h at 45 °C. The synthetic mechanism of QH is demonstrated in Fig. 1a.

### Chain Extension of Hemicelluloses

Molecular chain extension of hemicelluloses was obtained using ECH as crosslinking agent. In a typical method, 0.33 g of QH was dissolved in 10 mL of distilled water with mechanical stirring at 60 °C for 30 min. Sodium hydroxide solution (w%, 50%) was added into the solution to adjust pH to 13, followed by 5 mL of ECH. Then the mixture was stirred at 60 °C for 5 h to complete the crosslinking reaction. The resulting CQH were thoroughly washed with ethanol and filtered off, air dried for 12 h and further dried in an oven for 24 h at 45 °C. Figure 1b shows the synthetic mechanism of chain extension.

### Preparation of Composite Hydrogels

The composite hydrogels were prepared between CQH and AA by free radical graft copolymerization in the presence of crosslinking agent (MBA) and a redox initiator system (APS/TEMED). In a typical procedure of hydrogel preparation, CQH (0.1 g) were first dissolved in 10 mL of distilled water in a three-necked flask with vigorous mechanical stirring at 60 °C for 1 h, and then the solution was cooled to ambient temperature. 0.05 g of APS and 0.05 mL of TEMED as an initiator system were added into the CQH solution during continuous purge with gaseous N_2_, and the mixture was stirred for 10 min to generate radicals in nitrogen gas atmosphere. Variable amounts of AA (0.8–2.0 g) and crosslinking agent MBA (0.005–0.025 g) were added, and the mixture solution continued to be stirred at ambient temperature for 2 h under nitrogen gas atmosphere. After that, the mixture solution was transferred into a centrifuge tube (10 mL), and the crosslinking reaction was allowed to proceed at 65 °C for 24 h without mechanical stirring under nitrogen gas atmosphere. Finally, the hydrogels were carefully removed from the centrifuge tubes to further test the properties of hydrogels. Figure 1c shows the synthetic mechanism of hydrogels, and the composition of the fabricated hydrogels is shown in Table 1.

### FT-IR Spectroscopy

FT-IR spectra of the samples were obtained using a Thermo Scientific Nicolet iN 10 FT-IR microscope with an FT-IR spectrometer from 4000 to 650 cm^−1^ at a resolution of 4 cm^−1^ and 128 scans per sample.

### Swelling Capacity Measurements

The freeze-dried composite hydrogels were weighed and then immersed into distilled water to test their swelling capacity at room temperature. The swollen hydrogels were taken out and filtered with a nylon fabric bag filter for 20 min until no free water dripped, then weighed at regular intervals until the composite hydrogels reached the swelling equilibrium. The equilibrium water absorption was calculated using the following equation:


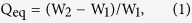


where Q_eq_ is the equilibrium water absorption, which is defined as grams of water per gram of sample. W_1_ and W_2_ are the mass of hydrogel samples before and after swelling, respectively.

### Morphology of Composite Hydrogels

The sectional structures of the composite hydrogel were measured by a SEM (HitachiS-3400NII) to observe its internal microstructure. Images were obtained that were dependent on the feature being traced.

### Mechanical Test

The composite hydrogels were shaped to cylindrical samples, and compression stress-strain tests were recorded using a compressive tester (CTM6503, Shenzhen SANS Technology stock Co., Ltd. China) at a compression speed of 2 mm min^−1^.

### Preosteoblast Culture

The hydrogels were sterilized by soaking with ethanol, and they were cut into cylindrical slices with a thickness of about 0.1 cm. Then the hydrogels were transferred to the 24-well plastic culture plates. The preosteoblasts were extracted from the calvaria of mus musculus. The dye was obtained using 1 mM calcein AM and 2 mM ethidium homodimer-1 solutions. Preosteoblasts, with a cell density of 2 × 10^5^ cells cm^−3^, were seeded onto the hydrogels in an incubator for 24 h and 36 h, respectively. The morphologies of preosteoblasts were measured by a SEM. The cells were stained with dye and then observed by a fluorescence microscope.

## Conclusion

Composite hydrogels were prepared by crosslinking AA monomers in the presence of the extended molecular chains of hemicelluloses. This new chain extension strategy significantly improved the mechanical performance of hydrogels. Abundant folds and lamellar structures in the hydrogels improved the swelling capacity. Different weight ratios of AA/CQH and MBA/CQH yielded swelling ratios that ranged from 100 to 1405 g/g in distilled water. Higher AA/CQH or MBA/CQH weight ratio resulted in higher crosslinking density and higher compression modulus, which would reduce the swelling capacity of hydrogels. The fabricated hydrogels showed good biocompatibility and preosteoblasts could adhere, spread, and proliferate well on the composite hydrogels. These results suggest that these fabricated hemicelluloses-based hydrogels have promising potential applications in many fields. Hemicelluloses-based hydrogels show excellent swelling performance and could be used as a soil and water humectant. Biocompatibility assay properties may allow use of hydrogels in cell culture and medicine delivery systems. Overall, the hemicelluloses-based hydrogels have a wide potential range of applications as environmentally friendly materials.

## Additional Information

**How to cite this article**: Qi, X.-M. *et al*. Enhanced mechanical performance of biocompatible hemicelluloses-based hydrogel via chain extension. *Sci. Rep.* **6**, 33603; doi: 10.1038/srep33603 (2016).

## Figures and Tables

**Table 1:** Composition of the fabricated hydrogels.

Sample	CQH(g)	AA/CQH(g/g)	MBA/CQH(g/g)
H-0.8	0.1	8	0.10
H-1.0	0.1	10	0.10
H-1.2	0.1	12	0.10
H-1.6	0.1	16	0.10
H-2.0	0.1	20	0.10
G-0.005	0.1	10	0.05
G-0.015	0.1	10	0.15
G-0.020	0.1	10	0.20
G-0.025	0.1	10	0.25

**Table 2:** Water content changes of hydrogels before and after storage for a week.

Sample code	Change of water content (%)
H-0.8	6.28
H-1.0	8.15
H-1.2	9.87
H-1.6	14.61
H-2.0	16.38
